# Protein Kinase D Interacts with Neuronal Nitric Oxide Synthase and Phosphorylates the Activatory Residue Serine^1412^

**DOI:** 10.1371/journal.pone.0095191

**Published:** 2014-04-16

**Authors:** Lucía Sánchez-Ruiloba, Clara Aicart-Ramos, Lucía García-Guerra, Julia Pose-Utrilla, Ignacio Rodríguez-Crespo, Teresa Iglesias

**Affiliations:** 1 Instituto de Investigaciones Biomédicas “Alberto Sols”, Consejo Superior de Investigaciones Científicas-Universidad Autónoma de Madrid (CSIC-UAM), Madrid, Spain; 2 Departamento de Bioquímica y Biología Molecular I, Universidad Complutense de Madrid (UCM), Madrid, Spain; 3 CIBERNED, Centro de Investigación Biomédica en Red sobre Enfermedades Neurodegenerativas, Instituto de Salud Carlos III, Madrid, Spain; University of Illinois at Chicago, United States of America

## Abstract

Neuronal Nitric Oxide Synthase (nNOS) is the biosynthetic enzyme responsible for nitric oxide (·NO) production in muscles and in the nervous system. This constitutive enzyme, unlike its endothelial and inducible counterparts, presents an N-terminal PDZ domain known to display a preference for PDZ-binding motifs bearing acidic residues at -2 position. In a previous work, we discovered that the C-terminal end of two members of protein kinase D family (PKD1 and PKD2) constitutes a PDZ-ligand. PKD1 has been shown to regulate multiple cellular processes and, when activated, becomes autophosphorylated at Ser^916^, a residue located at -2 position of its PDZ-binding motif. Since nNOS and PKD are spatially enriched in postsynaptic densities and dendrites, the main objective of our study was to determine whether PKD1 activation could result in a direct interaction with nNOS through their respective PDZ-ligand and PDZ domain, and to analyze the functional consequences of this interaction. Herein we demonstrate that PKD1 associates with nNOS in neurons and in transfected cells, and that kinase activation enhances PKD1-nNOS co-immunoprecipitation and subcellular colocalization. However, transfection of mammalian cells with PKD1 mutants and yeast two hybrid assays showed that the association of these two enzymes does not depend on PKD1 PDZ-ligand but its pleckstrin homology domain. Furthermore, this domain was able to pull-down nNOS from brain extracts and bind to purified nNOS, indicating that it mediates a direct PKD1-nNOS interaction. In addition, using mass spectrometry we demonstrate that PKD1 specifically phosphorylates nNOS in the activatory residue Ser^1412^, and that this phosphorylation increases nNOS activity and ·NO production in living cells. In conclusion, these novel findings reveal a crucial role of PKD1 in the regulation of nNOS activation and synthesis of ·NO, a mediator involved in physiological neuronal signaling or neurotoxicity under pathological conditions such as ischemic stroke or neurodegeneration.

## Introduction

Nitric oxide synthases (NOSs) are the enzymes responsible for ·NO production, a biological signaling molecule involved in the control of cardiovascular, immune and nervous system physiology [1]. Neuronal NOS (nNOS), is larger than both its endothelial (eNOS) and inducible (iNOS) counterparts, mostly due to a ∼300 amino acid N-terminal extension containing a PDZ domain (residues 14-105) [2], [3]. The association of this N-terminal sequence with other neuronal proteins determines nNOS enrichment at post-synaptic densities [4], [5]. Peptide library as well as yeast two-hybrid screens revealed that the PDZ module of nNOS displays a clear binding preference for cellular proteins with C-terminal acidic amino acids at -2 and -3 positions. In fact, proteins with a -Gly-(Asp/Glu)-X-Val C-terminus were proposed as tight binders of nNOS PDZ domain [6], [7]. Soon afterwards, a protein referred to as CAPON (C-terminal PDZ ligand of nNOS), displaying a C-terminal -Glu-Ile-Ala-Val motif and highly enriched in the brain was reported to bind to the PDZ domain of nNOS [8]. In a similar fashion, the acidic C-terminus of other neuronal proteins such as melatonin receptor (-Val-Asp-Ser-Val), phosphofructokinase-M (-Glu-Ala-Ala-Val) and NIDD (-Glu-Asp-Ile-Val) have been reported as ligands of the PDZ domain of nNOS [9]–[11]. In addition, the nNOS beta hairpin that extends the preformed PDZ domain mediates the formation of PDZ/PDZ dimers of nNOS/PSD-95 and nNOS/α1-syntrophin in neuronal cells [12], [13]. The postsynaptic density protein PSD-95 binds to the C-terminus of ionotropic N-Methyl-D-Aspartate (NMDA)-type of glutamate receptors (NMDARs) through PDZ1 and to nNOS through PDZ2 hence forming a ternary complex in neurons [14], [15]. Therefore, nNOS activation is enhanced after physiological or pathological NMDARs stimulation leading to ·NO production [16]–[18]. We have previously reported that in cortical neurons and brain, NMDARs also associate with kinase D interacting substrate of 220-kDa (Kidins220) [19], a protein also known as ankyrin-repeat rich membrane spanning (ARMS). Kidins220/ARMS is a neuronal enriched transmembrane protein identified as the first substrate of protein kinase D1 (PKD1) [20] and as a downstream effector of neurotrophin receptors [21].

Protein kinase D1 (PKD1) belongs to a family of phorbol ester/diacylglycerol-stimulated Ser/Thr kinases constituted by two additional members, PKD2 and PKD3 [22]. PKDs play multiple roles in different cell types and tissues, from primary cellular functions such as protein traffic, adhesion, migration, proliferation, survival and death, to complex processes such as immune regulation, cardiac hypertrophy, angiogenesis and cancer [22]. In addition, PKD1 has been involved recently in specific neuronal functions such as axon formation, sorting of dendritic proteins and dendritic arborization [23]–[25]. All PKD isoforms bear a cysteine-rich domain (CRD) that binds diacylglycerol and phorbol esters, an autoinhibitory pleckstrin homology domain (PH), followed by the catalytic domain [22]. Importantly, we discovered a unique distinctive type I PDZ-binding sequence or PDZ-ligand at the very C-terminal end of PKD1 and PKD2 that is absent in PKD3 [26]. In PKD1, kinase activation results in autophosphorylation of Ser^916^ located at -2 position within its PDZ-binding motif (-Val^915^-Ser^916^-Ile^917^-Leu^918^), which in turn controls Kidins220/ARMS transport and localization at the neuronal plasma membrane [26], [27]. These previous results led us to propose a model where the negative charge of the incorporated phosphate at this position in active PKD1 could mimic an acidic residue that could change the binding affinity of its PDZ-ligand for different PDZ proteins, regulating this way Kidins220/ARMS traffic [26]. Knowing that nNOS and PKD are spatially enriched in postsynaptic densities and dendrites, and that nNOS PDZ domain binds preferentially PDZ-ligands bearing acidic residues at -2 position, we hypothesized that the phosphorylated PDZ-binding motif of active PKD1 could be a bona-fide binding partner for the PDZ domain of nNOS. Herein, we have explored whether PKD1 activation could result in a direct interaction with nNOS and also if nNOS could be a substrate for PKD1, analyzing the functional consequences. Our studies show that PKD1 activation enhances its association with nNOS and favors their subcellular colocalization. However, contrary to our initial hypothesis, this association is independent of its PDZ-ligand but depends on the PH domain of PKD1. In addition, we demonstrate that PKD1 activates nNOS by phosphorylating the activatory residue Ser^1412^, leading to increased ·NO production, hence establishing a novel role of PKD in the regulation of ·NO synthesis.

## Materials and Methods

### Ethics Statement

Animal procedures were approved by “Consejo Superior de Investigaciones Científicas” - CSIC Ethics Committee and performed in compliance with European Directive 2010/63/EU. Animals used were kept to a minimum, they were sacrificed by deep anesthesia, and all efforts were made to minimize suffering.

### Cell Lines, Reagents and Antibodies

HEK293T, COS-7, and PC12 cells were obtained from American Type Culture Collection ATCC (Manassas, VA, USA). Phorbol-12, 13-dibutyrate (PDBu), 8-Br-cGMP, L-N^G^-nitroarginine methyl ester (L-NAME), N-Methyl-D-aspartate (NMDA), glycine, cytosine β-D-arabino furanoside (AraC), poly-L-lysine, L-laminin, Protein A/G-Sepharose, 2′,5′-ADP–Sepharose, adenosine 2′(3′)-monophosphate mixed isomers, DNA single stranded from salmon testes for hybridization, and 5-Bromo-4-chloro-3-indolyl β-D-galactopyranoside (X-Gal) and 4,5-Diaminofluorescein diacetate (DAF2-DA) were from Sigma Co. (St. Louis, MO, USA). Nerve growth factor was from Alexis Corp. (San Diego, CA, USA). Ni-NTA resin was from Qiagen (Chatsworth, CA, USA). L-Arginine and Gö6976 were purchased from Calbiochem (Merck Millipore, Darmstadt, Germany). [γ^32^P]-ATP (370 MBq/ml) was from PerkinElmer, Inc. (Boston, MA, USA). Mouse monoclonal anti-Myc, anti-GST and rabbit polyclonal antibodies recognizing total PKD1/2 and phospho-Ser^916^ were from Cell Signaling Technology (Beverly, MA, USA). Anti-β-tubulin I monoclonal antibody was purchased from Sigma and rabbit polyclonal anti-neuronal specific enolase (NSE) from ICN Biomedicals (Costa Mesa, CA, USA). We produced an antibody against nNOS immunizing rabbits with purified rat nNOS following standard procedures. Rabbit polyclonal anti-nNOS-phospho-Ser^1412^ was purchased from Upstate-Merck Millipore (EMD Millipore Corporation, Billerica, MA, USA). Mouse monoclonal antibody recognizing total VASP and rabbit polyclonal antibody anti-VASP-phospho-Ser^239^ were from Santa Cruz Biotechnology (Santa Cruz, CA, USA). Rabbit polyclonal anti-GFP was obtained from Invitrogen-Life Technologies (Carlsbad, CA, USA). Horseradish peroxidase-conjugated anti-rabbit and anti-mouse secondary antibodies were from General Electric (Fairfield, CT, USA). Oligonucleotide primers were from Invitrogen-Life Technologies (Carlsbad, CA, USA). All other reagents were from standard suppliers or as indicated in the text.

### Identification of PKD1-phosphorylated residue in nNOS by mass spectrometry or MALDI TOF/TOF

*In vitro* kinase reactions after phosphorylating nNOS by a recombinant protein containing the active catalytic domain of PKD1 fused to GST (GST-PKD1-cat) were digested with trypsin and analyzed by HPLC followed by MALDI TOF/TOF and peptide fragmentation and *de novo* sequencing in the Proteomic Studies Unit (Unidad de Proteómica; Facultad de Farmacia Parque Científico de Madrid, Universidad Complutense de Madrid, Madrid, Spain) following standard procedures. MALDI-TOF MS analysis was performed in a 4800 Proteomics Analyzer MALDI-TOF/TOF mass spectrometer (Applied Biosystems, MDS Sciex, Toronto, Canada). The MALDI-TOF/TOF operated in positive reflector mode with an accelerating voltage of 20000 V. Selected peptides, were subjected to MS/MS sequencing analyzes using the 4800 Proteomics Analyzer (Applied Biosystems, Framingham, MA). Suitable precursors from the MS spectra were selected for MS/MS analysis with CID on (atmospheric gas was used) 1 Kv ion reflector mode and precursor mass Windows ± 4 Da. The plate model and default calibration were optimized for the MS/MS spectra processing. De novo sequencing from fragmentation spectra of peptides was performed using *De novo* tool software (Applied Biosystems), tentative sequences were manually checked and validated.

### Yeast two hybrid screens

We used plasmids containing GAL4 binding domain that were confronted with plasmids containing the GAL4 activation domain as previously described [28]. Double transformants were plated in Leu^−^/Trp^−^/His^−^SD plates in the presence of 12 mM 3-amino triazole (TDO plates) as well as in Leu^−^/Trp^−^/His^+^. Interacting proteins expressed within the same yeast resulted in colonies that could rescue growth in the absence of His. These colonies were subsequently screened in the X-Gal assay. Blue colonies corresponded to a positive interaction whereas white colonies corresponded to absence of interaction. The complete PKD1 active catalytic domain (Gly-557 to Leu-918) or shorter C-terminal sequences (Pro-591 to Leu-918) containing the PDZ-binding motif of wild-type PKD1 or the phospho-mimetic mutant PKD1-Ser^916^Glu (PKD1^S916E^, described in Sanchez-Ruiloba et al. [26]) were PCR-amplified using primers carrying NdeI/EcoRI sites and subcloned into pGBKT7, in frame with the DNA-binding domain of GAL4. PKD1 baits were used to perform one to one yeast two hybrid assays against two nNOS constructs that were PCR-amplified using primers carrying EcoRI/SalI sites and subcloned into pGAD, in frame with the activation domain of GAL4: one shorter including the nNOS PDZ domain (aa 1–102) and a longer one (aa 1–131) that includes the C-terminal extension peptide of the nNOS PDZ domain that represents a relatively independent structural unit in mediating the interaction between nNOS and PDZ domain-containing proteins including PSD-95 and α1-syntrophin [12]. As controls both nNOS constructs were confronted with α1-syntrophin: the long nNOS construct as positive control and the short construct that lacks of the β-hairpin “finger” as a negative control. The C-terminus of rat CAPON (sequence ELGDSLDDEIAV) was cloned in the pGBT9 plasmid between the EcoRI and SalI sites.

### Cell culture and transfection

HEK293T or COS-7 cells were cultured in Dulbecco's modified Eagle's medium (DMEM; Invitrogen-Life Technologies; Carlsbad, CA, USA), supplemented with 10% (v/v) foetal calf serum, and 2 mM glutamine at 37°C in a humidified atmosphere containing 5% CO_2_. HEK293T cells were seeded at 60% confluence for transfection using Lipofectamine2000 reagent (Invitrogen-Life Technologies; Carlsbad, CA, USA), according to the manufacturer's specifications, and collected for processing 48 h later. Cell were transfected with empty vector pEFBOS-GFP or containing GFP fused to PKD1 wild-type (PKD1), kinase-inactive (the single mutant Asp^733^Ala; PKD1ki), constitutively active (the double mutant Ser^744/748^Glu; PKD1ca), PDZ-ligand mutants (PKD1-Ser^916^Glu/PKD1^S916E^; PKD1-Ser^916^Ala/PKD1^S916A^, or PKD1 lacking its PDZ-ligand/PKD1^ΔSIL^) and deletion mutants lacking the PH-domain (PKD1^ΔPH^) or the CRD domain (PKD1^ΔCRD^) that have been used previously [26], [29], [30]. Expression vectors for Myc-tagged wild-type rat nNOS (nNOS) and the point mutant nNOS-Ser^1412^Ala (nNOS^S1412A^) were kindly provided by Dr. G. A. Rameau and Dr. E. B. Ziff [31]. When required, HEK293T cells were treated with PDBu (200 nM) for 15 min, 8-Br-cGMP (100 µM) for 30 min or L-NAME (100 µM) for 24 h, as specified in the text. PC12 cells were cultured at 37°C in Dulbecco's modified Eagle's medium (DMEM; Invitrogen-Life Technologies; Carlsbad, CA, USA) supplemented with 7.5% fetal calf serum, 7.5% horse serum, and 2 mM glutamine in a humidified atmosphere containing 5% CO_2_. Cells were treated with nerve growth factor (75 ng/ml) for 2 days post-transfection. For transfection and immunofluorescence, HEK293T and PC12 cells were seeded at 50–60% confluence on poly-L-lysine (10 µg/ml)-coated glass coverslips. Cells were transfected as above and 48 h later cells were treated with PDBu (200 nM) for 15 min, fixed and processed for immunofluorescence.

### Cultures of primary cortical neurons

Cultures of dissociated E19 rat cortical neurons were prepared from the cerebral cortex of 19-day-old Wistar rat embryos as described [26]. Rats were obtained from the animal care facility at the Instituto de Investigaciones Biomédicas ‘Alberto Sols’ (CSIC-UAM, Madrid, Spain). Briefly, meninges were removed from the embryonic brains, and cortices were dissected. Tissue was resuspended in minimal essential medium (MEM; Invitrogen-Life Technologies; Carlsbad, CA, USA) complemented with 10% fetal calf serum, 10% horse serum, 0.6% glucose, 16 µg/ml gentamicin, and 2 mM glutamine. Cells were counted and seeded on laminin (4 µg/ml) and poly-L-lysine (10 µg/ml)-covered dishes at a final concentration of 5×10^5^ and incubated at 37°C in an atmosphere of 5% CO_2_. Neurons grown *in vitro* for 14 days (DIV14) were pretreated with Gö6976 (5 µM) for 1 h and left unstimulated or stimulated with the NMDAR agonist NMDA (50 µM) and its coagonist glycine (10 µM) for 5 min.

### Immunofluorescence and Confocal Microscopy

For immunofluorescence cells grown on coverslips were fixed for 10 min in 4% paraformaldehyde in phosphate-buffered saline at room temperature. After blocking (5% bovine serum albumin for 30 min) cells were incubated with the corresponding primary antibodies for 1 h at room temperature, and immunoreactivity was detected with the suitable fluorophore-conjugated secondary antibody before mounting in slides with ProLong (Invitrogen-Life Technologies; Carlsbad, CA, USA). Images are single sections of z-series acquiring each channel in a sequential mode using an inverted Zeiss LSM710 confocal microscope with a 63X/1.40 Plan-Apochromatic objective. Pictures were processed with ZEN 2009 light Edition (Carl Zeiss MicroImaging) and Adobe CS3 Extended (Adobe Systems Inc., CA) software.

### Protein extracts, immunoprecipitation and immunoblot analysis

Preparation of lysates and immunoprecipitation assays were performed as described previously [26]. Briefly, rat brain or cells were lysed in radioimmunoprecipitation assay buffer (25 mM Tris-HCl, pH 7.6, 1% Triton X-100, 1% sodium deoxycholate, 0.1% SDS, 150 mM NaCl, 2 mM EDTA, 2 mM dithiothreitol) with protease and phosphatase inhibitors for 30 min at 4°C, and lysates were then centrifuged for 20 min at 14,000 rpm. When needed, Myc-nNOS or Myc-nNOS^S1412A^ were immunoprecipitated with anti-Myc antibody during 4 h at 4°C. Equal amounts of total lysates or equivalent volumes of immunocomplexes were analyzed by SDS-PAGE followed by transfer to nitrocellulose filters and immunoblot. Membranes were blocked in TBST (20 mM Tris-HCl, pH 7.6, 137 mM NaCl, 0.05% Tween 20) plus 5% low-fat milk powder and incubated with the different primary and secondary antibodies in blocking solution and immunoreactive bands were visualized by enhanced chemiluminescence (ECL; PerkinElmer, Inc., Boston, MA, USA).

### Cloning and expression of full-length nNOS and the two independent heme-oxygenase and reductase domains

Using rat nNOS as a template, the N-terminal half of nNOS comprising the heme-oxygenase domain (residues 1 to 759) or the C-terminal half of nNOS comprising the reductase domain (residues 715 to 1429) were amplified and NdeI and XbaI sites were introduced at the 5′and 3′end respectively. The PCR bands were digested with NdeI/XbaI and ligated in the corresponding sites of 6His-pCWori [32] and verified by automated DNA sequencing. Each of the two halves of nNOS included the calmodulin binding sequence, since calmodulin binding assists protein folding and total yield [33]. Full-length nNOS expression and purification in this vector has been already described [34]. In general, protein expression and purification was performed in BL21 cells in coexpression with a calmodulin chloramphenicol-resistant pACYC plasmid as previously described [32]. Full-length nNOS and its reductase domain were purified using a Ni-NTA affinity resin followed by a 2′,5′-ADP sepharose whereas the heme-oxygenase was purified using only the Ni-NTA resin as previously described [32], [33].

### Cloning and expression of recombinant active catalytic domain of PKD1 fused to GST

The C-terminal region of PKD1 (Ser558-Leu918; PKD1cat) containing the full-length catalytic domain was amplified using as template pBS-PKD1 using oligonucleotides 5′ (5′-AAA AAG CAG GCT CCG GAT CCA ACT CAC ACA AAG ATA-3′) and 3′ (5′-AGA AAG CTG GGT TTT TGA CAG ATT AGA GGG GAT GGA-3′). The PCR product was cloned in pDONR201 by a recombination reaction with BP clonase (GATEWAY system, Invitrogen-Life Technologies; Carlsbad, CA, USA), to generate the construct pENTR-PKD1cat. After automated sequencing, PKD1cat was subcloned in pDEST15 using LR clonase. This vector for procaryotic expression generates PKD1cat fused to glutathione S-transferase (GST; GST-PKD1-cat) of approximate molecular weight of 65 kDa that was purified following standard methods and stored at −20°C. This protein is constitutively active since it lacks the regulatory autoinhibitory domain.

### Pull-down assays using recombinant pleckstrin homology domain of PKD1 fused to GST

Preparation of GST-PH domain of PKD and pull-down assays has been described previously [35]. Brain extracts or purified nNOS were incubated for 2 h at 4°C with either GST (control) or GST-PH fusion proteins pre-adsorbed onto glutathione-agarose beads and the presence of nNOS was analyzed by Western blot.

### *In Vitro* Kinase Assay

PKD or nNOS were immunoprecipitated from cultures of primary rat cortical neurons DIV14, and PKD phosphorylation activity was determined performing similar *in vitro* kinase assays as described previously [26]. nNOS phosphorylation by active catalytic domain of PKD1 (GST-PKD1-cat) was also analyzed using this type of assays. Briefly, PKD or nNOS immune-complexes or purified full-length nNOS or heme-oxygenase and reductase domains mixed with GST-PKD1-cat were resuspended in kinase buffer (30 mM Tris-HCl, pH 7.6, 10 mM MgCl_2_, and 2 mM dithiothreitol), and subjected to an *in vitro* kinase assay for 30 min at 30°C in the presence of 100 µM final concentration of [γ^32^P] ATP or non-radioactive ATP. Samples were analyzed by SDS-PAGE and Ponceau staining, autoradiography or immunoblot as indicated in the text.

## Results

### PKD1 interacts with nNOS through its PH domain but not its PDZ-ligand

**Figure 1:**
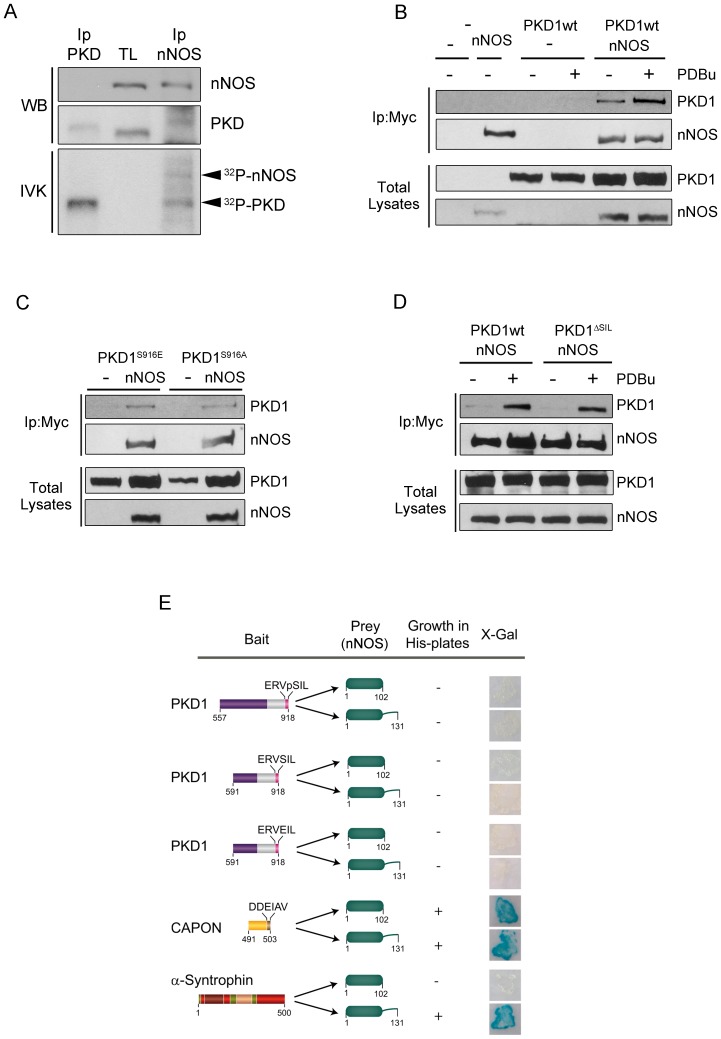
PKD1 association with nNOS is enhanced by kinase activation in a PDZ-ligand independent manner. (**A**) PKD and nNOS were immunoprecipitated from cultured primary rat cortical neurons DIV14. These immunoprecipitates were used to perform an *in vitro* kinase assay (IVK) in the presence of [γ-^32^P]-ATP before being resolved in SDS-PAGE gels together with neuronal total lysates (TL) and transferred to a nitrocellulose membrane. Filter was first incubated with specific antibodies to determine the presence of PKD or nNOS in the immunoprecipitates by Western blot analysis (WB). This method detected a signal that could correspond to PKD in nNOS immunoprecipitates. Note that PKD showed a slower migration after IVK compared to total lysates (TL) indicative of its hyperphosphorylated state. This membrane was then exposed to obtain an autoradiography image of the IVK. Autophosphorylated PKD was present both in PKD and nNOS immunoprecipitates (^32^P-PKD) and an additional radioactive band corresponding to nNOS was also detected. Radioactive bands and immunoblot signals matched completely after overlapping autoradiography and ECL films, indicative of the association of endogenous PKD with endogenous nNOS in primary cortical neurons DIV14. (**B**) HEK293T cells were transfected with Myc-nNOS (nNOS) and wild-type GFP-PKD1 (PKD1wt). Before lysis and 48 h after transfection, cells were untreated (−) or treated (+) with 200 nM PDBu for 15 min as indicated. Total lysates were subjected to immunoprecipitation with Myc antibody (Ip: Myc). Immunocomplexes were separated by SDS-PAGE and PKD1 and nNOS presence was analyzed by immunoblot using anti-GFP and anti-Myc antibodies, respectively. Expression levels of nNOS and PKD1wt in total lysates are also shown. Note that PDBu treatment enhances the formation of nNOS/PKD1 complexes. (**C**) A phospho-mimetic mutant GFP-PKD1-Ser^916^Glu (PKD1^S916E^) and a non-phosphorylatable mutant GFP-PKD1-Ser^916^Ala (PKD1^S916A^) within PKD1 PDZ-binding motif were transfected alone (-) or together with Myc-nNOS (nNOS). Total lysates were subjected to immunoprecipitation with Myc antibody (Ip: Myc). Immunocomplexes were separated by SDS-PAGE and PKD1 and nNOS presence was analyzed by immunoblot using anti-GFP and anti-Myc antibodies, respectively. Expression levels of nNOS and PKD1 mutants in total lysates are also shown. Note mutants in the PDZ-ligand of PKD1 are able to associate with nNOS similarly to PKDwt under non-stimulated conditions shown in panel B (**D**) Myc-nNOS (nNOS) was cotransfected into HEK293T cells together with wild-type GFP-PKD1 (PKD1wt) or a mutant where the PDZ-ligand had been deleted (PKD1^ΔSIL^), and 48 h after transfection cells were untreated (−) or treated (+) with PDBu as in panel B. Analysis of nNOS immunoprecipitates showed that PKD1/nNOS complexes are still formed after deletion of PKD1 PDZ-ligand. Representative images from three independent experiments are shown in panels A, B, C and D. (**E**) Active catalytic domain of PKD1 (aa 557–918), presenting phosphorylated Ser^916^ in its PDZ-ligand (ERVpSIL), or two shorter C-terminal fragments containing the non-phosphorylated PDZ-binding motif of wild-type PKD1 (ERVSIL) or a phospho-mimetic mutant PKD1-Ser^916^Glu (ERVEIL) were cloned in pGBKT7 and used as baits in a yeast two hybrid assay using as prey the PDZ domain of nNOS (without or with the β-hairpin “finger” extension) cloned in pGAD. A positive interaction was detected by the ability of the yeasts to grow in the absence of histidine and to metabolize the X-Gal substrate. No direct interaction between any of PKD1 baits tested was found. The C-terminus of CAPON was used as a positive control of binding to both nNOS constructs whereas α-syntrophin was used as a control of protein known to bind to nNOS only in the presence of the β-hairpin.

Proteins known to bind to nNOS PDZ domain must present a hydrophobic amino acid such as Val, Leu or Ile at the final position together with an acidic residue at position -2 or - 3 [6], [7]. The C-terminus of PKD1 possesses a -VSIL motif in which the Ser residue (Ser^916^) becomes autophosphorylated in the active enzyme [26], [27]. Therefore, we reasoned that the negative charge of the phosphate incorporated at Ser^916^ in active PKD1 could convert this domain in a bona-fide PDZ-binding motif for the PDZ domain of nNOS. To check this idea, and given that nNOS and PKD are spatially enriched in postsynaptic densities and dendrites, we first examined the possible association of both enzymes in mature neurons performing co-immunoprecipitation assays. Cultured primary rat cortical neurons grown *in vitro* for 14 days (DIV14) were lysated and PKD and nNOS were immunoprecipitated using specific antibodies (Figure 1A). These immunoprecipitates were used to perform an *in vitro* kinase assay (IVK) in the presence of [γ-^32^P]-ATP before being resolved in SDS-PAGE gels and transferred to a nitrocellulose membrane. This filter was first subjected to Western blot analysis to examine the presence of PKD or nNOS in the immunoprecipitates. As shown in figure 1A, nNOS was not present in PKD immunoprecipitates while a band that could correspond to PKD was detected in nNOS immunoprecipitates. It is noticeable that immunoprecipitated PKD subjected to IVK migrated more slowly, indicating its hyperphosphorylated state compared to its signal in neuronal total lysates. Next, this membrane was exposed to obtain an autoradiography image (Figure 1A, IVK, bottom panel). We clearly observed a radioactive band corresponding to autophosphorylated PKD in both immunoprecipitates. Importantly, nNOS immunoprecipitates showed an additional radioactive band of an apparent molecular weight similar to that of nNOS (160 kDa). When this autoradiography was overlapped with immunoblots developed with PKD or nNOS antibodies we could observe that both signals matched completely. This result demonstrates that endogenous nNOS is able to co-immunoprecipitate endogenous PKD from mature neuronal lysates and suggests that nNOS could be a PKD substrate.

To further support PKD-nNOS association, we analyzed if we could detect the association of PKD and nNOS by co-immumoprecipitation and Western blot using epitope-tagged versions of both proteins transfected into mammalian cells. Here, we also examined how PKD1 activation could affect their association. HEK293T cells were transfected with Myc-nNOS together with GFP-PKD1 wild-type (PKD1wt) and 48 h later were left untreated or treated with the phorbol ester PDBu in order to activate PKD (Figure 1B). After immunoprecipitating nNOS using an anti-Myc antibody, the presence of GFP-PKD1 in the immunocomplexes was assessed detecting GFP signal by immunoblot. These experiments showed that PKD1wt was present in nNOS immunoprecipitates and that this result was clearly enhanced after PDBu treatment (Figure 1B). This result confirmed the association of these two enzymes and indicated that PKD1 activation potentiates the formation of PKD1/nNOS complexes.

Because PKD activation leads to Ser^916^ autophosphorylation within its PDZ-binding motif, we further examined the contribution of this phosphorylation to the association of PKD1 with nNOS. To this end, HEK293T cells were cotransfected with Myc-nNOS together with a phospho-mimetic mutant GFP-PKD1-Ser^916^Glu (PKD1^S916E^) or a non-phosphorylatable mutant GFP-PKD1-Ser^916^Ala (PKD1^S916A^). Co-immunoprecipitation analysis performed as above showed that mutation of this residue did not alter the association of PKD1 with nNOS under basal conditions and in the absence of PDBu (Figure 1C). Importantly, the association of these two PKD1 mutants to nNOS was comparable to that of their wt counterpart under resting non-stimulated conditions, indicating that mutations mimicking or abolishing phosphorylation of Ser^916^ were not regulating this process. Additional cotransfection experiments using a PKD1 mutant lacking its PDZ-ligand (PKD1^ΔSIL^) rendered a similar result in which PKD1 was able to co-immunoprecipitate with nNOS preferentially after kinase activation by PDBu treatment (Figure 1D). These results suggested that the PDZ-binding motif of PKD1 was dispensable for the formation of a complex with nNOS.

In order to complement these studies, and to definitely rule out the possible participation of PKD PDZ-ligand on its association with nNOS, we tested the putative interaction between the autophosphorylated C-terminus of PKD1 and nNOS PDZ domain performing a yeast two-hybrid assay. Initially, we employed the complete active catalytic domain of PKD1 (aa 557–918), presenting the autophosphorylated PDZ-stretch (-ERVpS^916^IL) at its very C-terminal end, and used it as bait to screen its binding to the PDZ domain of nNOS (Figure 1E). Contrary to our initial prediction, we found that this PKD1 construct failed to form a complex with either nNOS PDZ domain (residues 1–102) or an nNOS construct that included also the beta-hairpin motif (residues 1–131) (Figure 1E). We obtained a similar result using two shorter constructs of PKD1 displaying a wild-type non-phosphorylated motif (-ERVSIL) or a phospho-mimetic sequence (-ERVEIL) (Figure 1E). Control experiments showed that both nNOS constructs bound tightly to the PDZ-ligand of CAPON (Figure 1E), a protein with an acidic residue at the -3 position known to bind nNOS PDZ domain [8]. In addition, full-length α-syntrophin could associate only to the nNOS construct that included the beta-hairpin extension (Figure 1E), in agreement with the PDZ/PDZ domain interaction of these two molecules/proteins previously reported [12]. These results reflect that, albeit nNOS PDZ domain constructs used in this assay are functional, the presence of a negative charge on Ser^916^ at -2 position within PKD1 PDZ-ligand could not convey on this kinase the ability to bind to PDZ domain of nNOS. These data are also in agreement with immunoprecipitation experiments and further demonstrate that, contrary to our initial hypothesis, the PDZ-binding motif of PKD1 was dispensable for the association of the kinase with nNOS, even though there was a clear association between these two enzymes.

**Figure 2:**
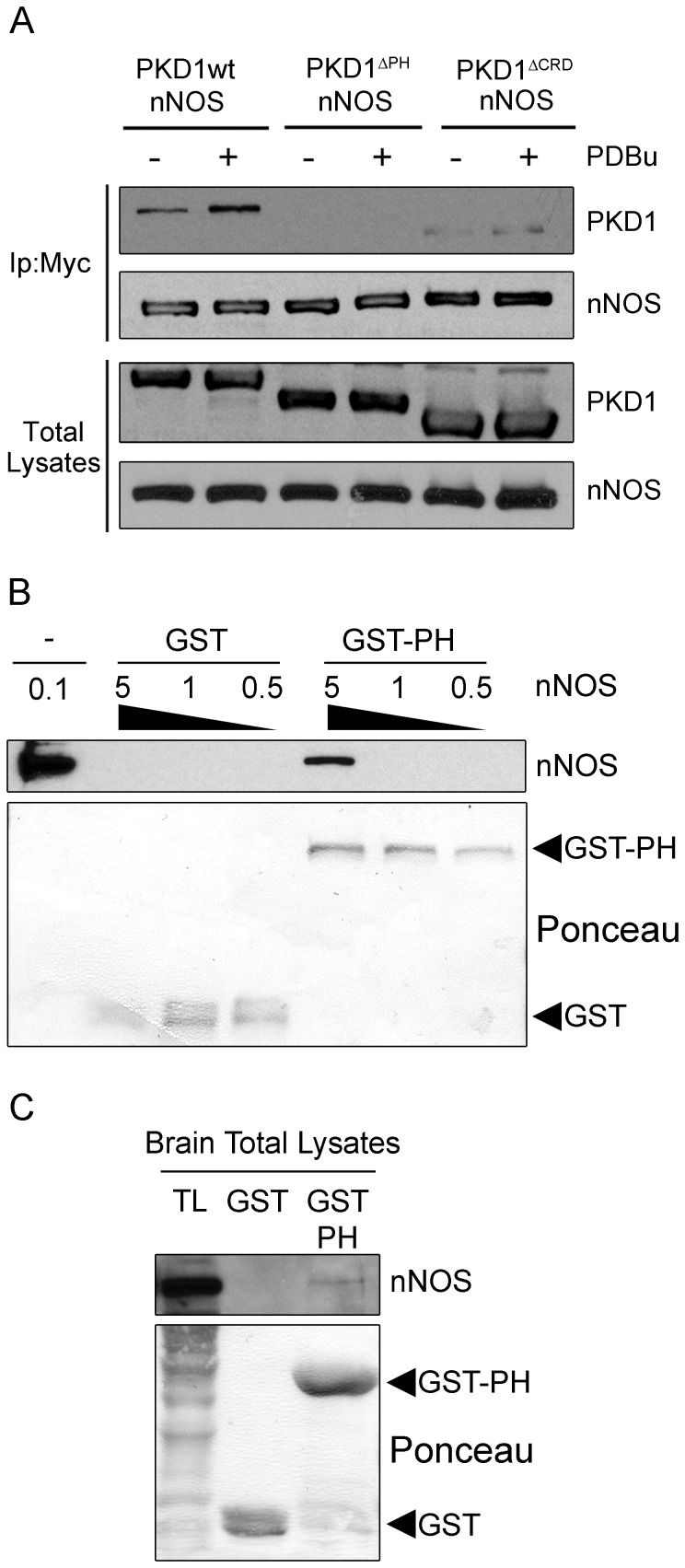
PKD1 PH domain mediates nNOS interaction. (**A**) HEK293T cells were transfected with Myc-nNOS (nNOS) together with wild-type GFP-PKD1 (PKD1wt) or mutants lacking the PH domain (PKD1^ΔPH^) or the cysteine-rich domain (PKD1^ΔCRD^), and treated (+) or not (−) with PDBu 48 h later. Total lysates were subjected to immunoprecipitation with Myc antibody (Ip: Myc) to immunoprecipitate nNOS. The presence of PKD1 and nNOS in immunocomplexes and total lysates was analyzed by immunoblot using anti-GFP and anti-Myc antibodies, respectively. Note that deletion of the PH domain in PKD1 hampers the formation of nNOS/PKD1 complexes. (**B**) Different concentrations of purified nNOS (0.5, 1 and 5 µg) were incubated with 8 µg of immobilized GST or GST-PKD-PH (GST-PH) proteins. Pull-down complexes were run together with 0.1 µg of purified nNOS in SDS-PAGE gels and nNOS was detected by Western blot. (**C**) Rat brain extracts (500 µg) were incubated with GST or GST-PH and the presence of nNOS in pull-down samples and in brain total lysates (TL) was determined by Western blot as above. Loading of proteins is shown by Ponceau staining. Results are representative of three independent experiments.

We continued examining the participation of other PKD1 domains that could be mediating PKD and nNOS association transfecting into mammalian cells GFP-PKD1 mutants where the PH or CRD domains had been deleted (PKD1^ΔPH^ and PKD1^ΔCRD^, respectively). HEK293T cells transfected with these mutants together with nNOS for 48 h were untreated or treated with PDBu for 15 min to activate PKD1. Cellular lysates were immunoprecipitated with an anti-Myc antibody to detect PKD1 and nNOS co-immunoprecipitation. As shown in figure 2A, PKD1 without the CRD domain still associated to nNOS, however, nNOS/PKD1 complexes formation was absolutely hampered when PKD1 lacked its PH domain. This result is particularly important because we have shown that PKD1^ΔPH^ mutant is constitutively active and autophosphorylates at the PDZ-ligand Ser^916^ [36], and further supports the data obtained so far in yeast and mammalian cells, indicating again that phosphorylation of the PDZ binding motif is not involved in nNOS/PKD1 association. Given that PKD2 and PKD3 isoforms contain conserved PH domains, we also checked their possible association with nNOS performing transient transfections in HEK293T cells and coimmunoprecipitation analysis. Results showed that full-length PKD2 weakly interacted with nNOS (see Figure S1), whereas PKD3 did not (not shown), suggesting a higher preference of nNOS for binding to PKD1. Globally, from these experiments we can conclude that the presence of the PH domain of PKD1, but not its PDZ-ligand (phosphorylated or not), is absolutely required for the association of this kinase with nNOS.

Finally, in order to test whether PKD1 PH domain could be mediating a direct interaction with nNOS, we performed pull-down assays using recombinant GST-PH protein. Figure 2B shows that the PH domain of PKD1 alone was able to interact with purified nNOS. Furthermore, this domain also pulled-down nNOS from brain extracts (Figure 2C). These data indicate that PKD1 and nNOS interact directly through the PH domain of the kinase.

### PKD1 activation potentiates its colocalization with nNOS

**Figure 3:**
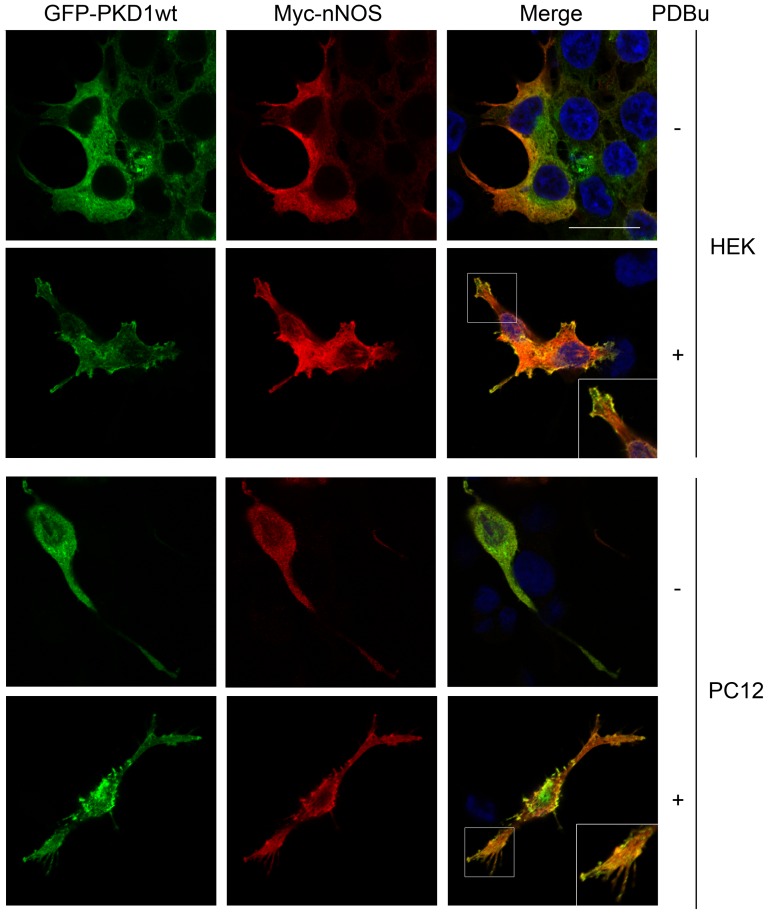
Activation of PKD1 increases its colocalization with nNOS. HEK293T and nerve growth factor treated PC12 cells were cotransfected with Myc-nNOS and wild-type GFP-PKD1 (GFP-PKD1wt). To activate PKD1, cells were left untreated or stimulated with PDBu for 15 min, immunostained using an anti-Myc antibody and analyzed by confocal microscopy. See that PKD1 activation enhances its colocalization with nNOS in both cell types. Results are representative of three independent experiments. Confocal microscopy images correspond to single sections. A magnified detail of the merge images in PDBu treated cells is depicted. *Scale bar*, 20 µM.

Depending on cell context and stimulation conditions PKD can be targeted to different intracellular locations such as the cytosol, plasma membrane, Golgi apparatus, or nucleus (for review, see [22]). In many cell types, including neural PC12 cells, PKD is mainly cytosolic and treatment with phorbol esters or receptor stimulation provokes a rapid recruitment of the enzyme to specific plasma membrane domains [29], [30], [37]. In addition, subcellular targeting of nNOS is also critical for the regulation of its function [4]. Given that PKD1 activation enhances its association with nNOS, we examined whether it could also promote their intracellular colocalization. To this aim, Myc-nNOS together with GFP-PKD1wt were transfected into HEK293T and nerve growth factor-treated PC12 cells. Two days after transfection cells were left untreated or stimulated with PDBu for 15 min to activate PKD1, then fixed and immunostained using an anti-Myc antibody, and analyzed by confocal microscopy. Immunofluorescence images from both cell types showed that under resting conditions PKD1 and nNOS presented a major cytoplasmic distribution and low colocalization (Figure 3). However, after phorbol ester stimulation PKD1 translocated to certain subdomains of the plasma membrane where it significantly co-localized with nNOS (Figure 3). This result reinforces that PKD1 activation potentiates its association and subcellular colocalization with nNOS.

### PKD1 phosphorylates nNOS at activatory Ser^1412^ *in vitro* and in live cells

**Figure 4:**
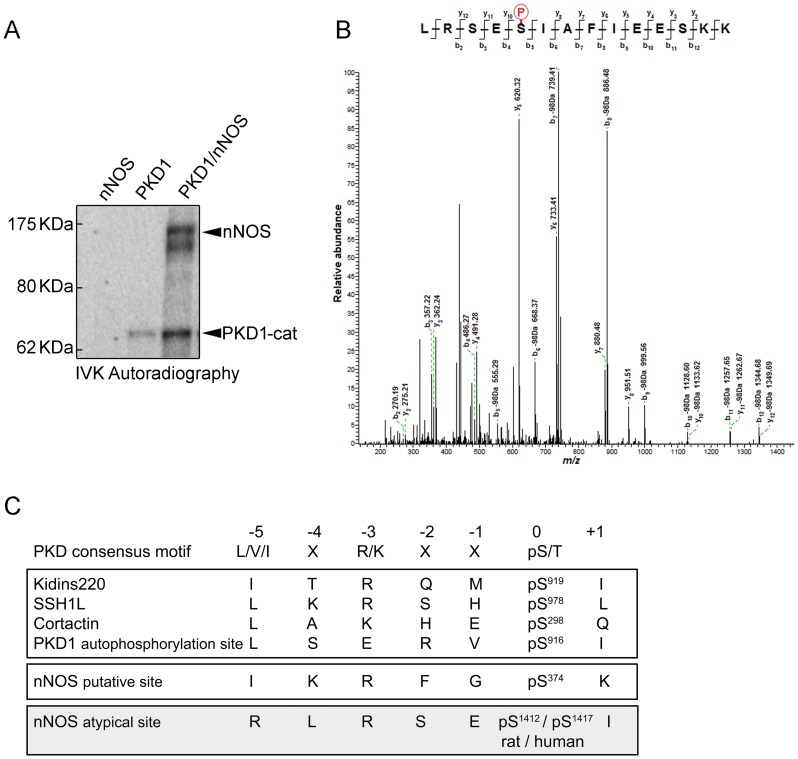
Identification of Ser^1412^ in nNOS as the unique site targeted by PKD1 phosphorylation. (**A**) Purified full-length rat nNOS (1429 amino acids, accession number P29476) was phosphorylated by purified active catalytic domain of PKD1 (PKD1-cat) in an *in vitro* kinase assay (IVK) using [γ-^32^P]-ATP. The image shows a representative IVK autoradiography out of three independent assays performed. (**B**) nNOS phosphorylated *in vitro* by PKD1 as in (A), but using non-radioactive ATP, was digested with trypsin, and the resulting peptides analyzed by HPLC coupled to MALDI-TOF/TOF. The MS/MS spectra of the tryptic nNOS peptide ^1408^LRSEpSIAFIEESKK^1421^ (Mass, 1715,851 Da) is shown. The “y-ion fragment series” and the “b-ion fragment series” are indicated on the top. Fragmentation of the precursor reveals unambiguously that Ser^1412^ is the phosphorylation site. No other phosphopeptides could be detected among the over 200 peptides resolved by HPLC coupled to MALDI-TOF/TOF analysis. (**C**) Consensus motif for PKD phosphorylation and sites of phosphorylation in several PKD substrates (Kidins220; Slingshot-SSH1; Cortactin) and PKD1 C-terminal autophosphorylation motif. The phosphorylatable Ser (pS) is at position P(0), residue at P(-3) is typically occupied by a basic residue (Arg/Lys) and a hydrophobic amino acid is characteristic of P(-5) (preferentially Leu/Val/Ile). Although nNOS Ser^374^ fulfilled the criteria of putative PKD consensus phosphorylation motif, it was not found to be phosphorylated by the kinase. Instead C-terminal nNOS Ser^1412^ was identified as a phosphorylated site by mass spectrometry and represents an atypical consensus sequence for PKD since residue at P(-5) is occupied by an Arg.

Our initial *in vitro* kinase and immunoblot analysis suggested that immunoprecipitated nNOS from neuronal extracts could be phosphorylated by PKD (Figure 1A, IVK). Sequence analysis of nNOS revealed that the heme-oxygenase domain displays one consensus site for PKD1 phosphorylation (IKRFG-pS**^374^**-K) [38]. Therefore, we further investigated whether nNOS was a PKD substrate performing *in vitro* kinase assays using radioactive [γ^32^P]-ATP and purified enzymes. As shown in figure 4A, recombinant full-length nNOS incubated with the purified active catalytic domain of PKD1 (PKD1-cat) rendered a clear radioactive band at 160 kDa, indicative of nNOS phosphorylation. Autophosphorylated PKD1 catalytic domain was also detected as a radioactive band of 65 kDa (Figure 4A).

In order to identify the residues phosphorylated by PKD1 within nNOS, a similar *in vitro* kinase reaction, performed with non-radioactive ATP, was digested with trypsin and subsequently subjected to HPLC and peptide fragmentation by MALDI TOF/TOF (Figure 4B). Of the several hundred nNOS-derived peptides that were obtained, the only significant phosphopeptide that was clearly identified corresponded to LRSESIAFIEESKK (residues L1408-K1421 of rat nNOS - Accession number P29476; 1429 aa). *De novo* sequencing of an eluted tryptic peptide with a mass of 1715,85 Da revealed that it corresponded to sequence LRSE(pS)IAFIEESKK and the phosphorylated residue was unambiguously assigned to the Ser residue present at the fifth position (pS; b_5_ in Figure 4B). This analysis allowed us to identify accurately nNOS Ser^1412^ (Rat LRSE-pS^1412^-IAFIEESKK, residue that in human sequence corresponds to Ser^1417^) as the serine phosphorylated by PKD1 (Figure 4B). In this context, it must be mentioned that according to crystallographic data, Ser^1412^ is located within the nNOS C-terminal α-helix and its phosphorylation is known to activate the enzyme, inducing a conformational change that increases the NADPH-derived electrons from the reductase towards the heme-oxygenase domain [39].

Since *in silico* analysis of nNOS sequence did not predict this serine was in a consensus context for PKD1 phosphorylation, we compared the amino acid sequences of known phosphorylation sites within several other PKD1 substrates and searched for homologies with the one we had just identified (Figure 4C). PKD1 substrates typically present a hydrophobic residue such as Leu or Ile at -5 position, together with a basic residue such as Lys or Arg at -3 position [38]. Interestingly, C-termini of both PKD1 and nNOS partially fail to fully meet this requirement, since an acidic Glu residue is present at -3 position in PKD1 while a basic Arg residue is present at -5 position in nNOS. However, these C-terminal sequences are indeed bona fide PKD1 substrates. In the case of PKD1, C-terminal Ser^916^ can be not only autophosphorylated but also trans-phosphorylated by other active PKD1 molecules [26]. In the case of nNOS C-terminus, PKD1 phosphorylates Ser^1412^ both *in vitro* and in living cells as we demonstrate herein (see data below). Furthermore, various amino acids present at nNOS PKD1 phosphorylation sequence are identical to those present in other known PKD1 substrates. In addition to the conserved Arg residue at position -3, nNOS phosphorylation sequence displays a Ser at -2 position (as in the case of slingshot-SSH1 [40]) and a Glu at -1 position (as in the case of cortactin [41]) (Figure 4C).

**Figure 5:**
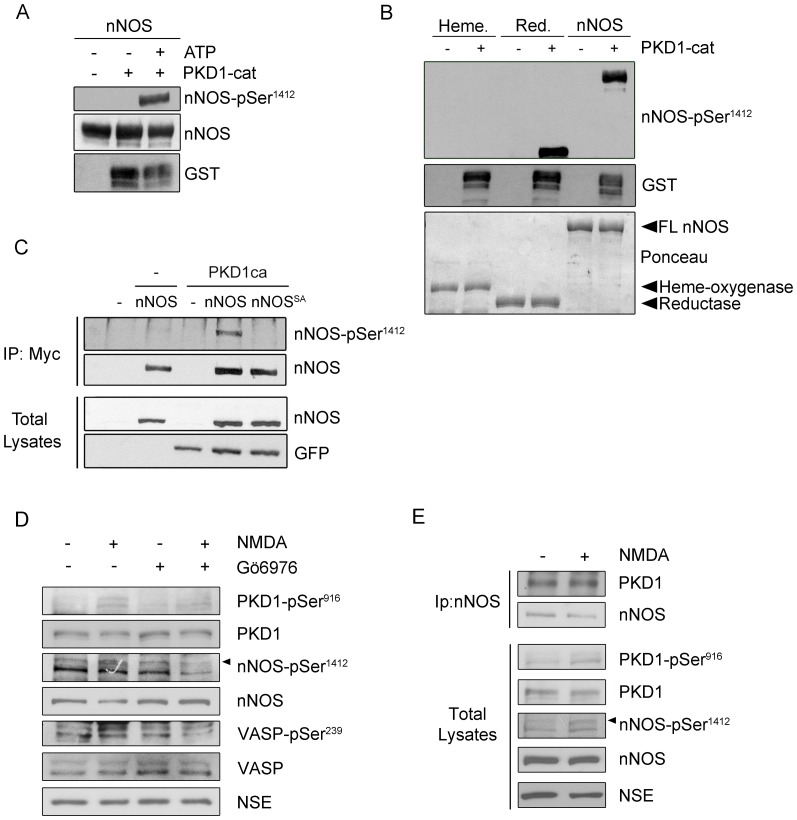
nNOS is phosphorylated by PKD1 at the activatory residue Ser^1412^ within the reductase domain. (**A**) Purified wild-type full-length nNOS was phosphorylated by purified active catalytic domain of PKD1 fused to GST (PKD1-cat active) by *in vitro* kinase assays (IVK) using non-radioactive ATP. nNOS phosphorylation by PKD1 at Ser^1412^ was detected by immunoblot using a phospho-specific antibody recognizing phospho-Ser^1412^ within nNOS reductase domain (nNOS-pSer^1412^). (**B**) *In vitro* phosphorylation of PKD1-cat occurs specifically at Ser^1412^ both when full-length nNOS or its reductase domain are used as substrates, but not when the heme-oxygenase domain is used as substrate. Phosphorylation was determined using anti phospho-Ser^1412^ antibodies. Loading of the different recombinant nNOS (full-length, or FL, heme-oxygenase or reductase domains) is shown by Ponceau staining. (**C**) HEK293T cells were cotransfected with either pEFBOS-GFP vector alone (−) or mutant active PKD1 (PKD1ca) and Myc-tagged nNOS or its phosphorylation deficient mutant Myc-nNOS-Ser1412Ala (nNOS^SA^). Two days later cells were lysed and total lysates were incubated with an anti-Myc antibody (IP: Myc). Detection of phosphorylated nNOS at Ser^1412^ or total nNOS in the immunocomplexes was determined by immunoblot analysis using a phosphospecific antibody (nNOS-pSer^1412^) or a total nNOS antibody. Levels of nNOS and GFP-PKD1ca in total lysates are also shown. (**D**) Primary cultures of rat cortical neurons grown *in vitro* for 14 days were untreated or treated with 50 µM NMDA plus 10 µM glycine (NMDA) for 5 min, pre-incubated or not for 1 h with the inhibitor Gö6976 (5 µM). Detection of active PKD (PKD-pSer^916^), total PKD, phosphorylated nNOS at Ser^1412^ or total nNOS in the lysates was determined by immunoblot analysis. Signal for the neuronal specific enolase (NSE) was used as loading control. Representative blots from three independent experiments are shown. (**E**) Primary cultures of rat cortical neurons grown *in vitro* for 14 days were untreated or treated with 50 µM NMDA plus 10 µM glycine (NMDA) for 5 min, pre-incubated or not for 1 h with the inhibitor Gö6976 (5 µM). Detection of active PKD (PKD-pSer^916^), total PKD, phosphorylated nNOS at Ser^1412^ or total nNOS in the lysates was determined by immunoblot analysis. Signal for the neuronal specific enolase (NSE) was used as loading control. (**E**) Endogenous nNOS was immunoprecipitated from cultured primary rat cortical neurons DIV14 untreated or treated with NMDA as above. These immunoprecipitates were analyzed for the presence of PKD and nNOS by Western blot. Total lysates from these neurons were run in parallel and the corresponding proteins were detected by the indicated antibodies.

In order to corroborate that Ser^1412^ was phosphorylated by PKD1 in nNOS we used a commercially available phospho-specific antibody recognizing this phospho-site (nNOS-pSer^1412^). We performed *in vitro* kinase assays as above followed by immunoblot analysis. The nNOS-pSer^1412^ antibody only detected purified nNOS when it had been pre-incubated with active PKD1 catalytic domain in the presence of ATP (Figure 5A). In addition we carried out a similar assay using the two independent domains to show that PKD1 was able to phosphorylate Ser^1412^ in the full-length protein and in the reductase domain (Figure 5B). To further validate our *in vitro* data and to test whether nNOS was also a substrate of PKD1 *in vivo* we transfected HEK293T cells with Myc-tagged wild-type nNOS (nNOS) or the non-phosphorylatable mutant nNOS-Ser^1412^Ala (nNOS^SA^) together with a constitutively active mutant of PKD1 fused to GFP (PKD1ca). Levels of ectopically expressed GFP-PKD1ca or mutated or wild-type Myc-nNOS were similar in all cellular total lysates. In agreement with our mass spectrometry results, after immunoprecipitating nNOS with anti-Myc antibodies we detected that Ser^1412^ was phosphorylated *in vivo* only in cells that had been cotransfected with constitutively active PKD1 (Figure 5C). Accordingly, no signal was detected when nNOS-Ser^1412^Ala mutant was used (Figure 5C). Given that glutamate stimulation of NMDAR in primary cultured cortical neurons results in nNOS Ser^1412^ phosphorylation [31] we finally examined whether PKD1 activation could occur downstream the activation of these type of glutamate receptors and control nNOS phosphorylation in this particular site (Figure 5D). Cortical neurons DIV14 were incubated with the NMDAR agonist NMDA and its co-agonist glycine for 5 min (named from now on as treatment with NMDA). Some neurons were pretreated for 1 h with Gö6976, an inhibitor that is frequently used to inhibit PKD [42], [43]. Importantly, immunoblot analysis of neuronal lysates showed increased levels of both active PKD phospho-Ser^916^ and nNOS phospho-Ser^1412^ after NMDAR stimulation, an effect that was significantly blocked by preincubation with the inhibitor (Figure 5D). Altogether our data show that PKD1 specifically phosphorylates Ser^1412^ in nNOS both *in vitro* and *in vivo*.

In figure 1A, we have already shown that endogenous PKD and nNOS specifically co-immunoprecipitated in lysates from mature cortical neurons in culture DIV14. Since PKD activation by PDBu increased its association with nNOS in transfected cells, we next examined whether this interaction could be also enhanced by NMDAR stimulation in neurons. We therefore analyzed PKD and nNOS co-immunoprecipitation under basal conditions or after stimulation with NMDA and found that NMDA treatment only slightly enhanced the association of both enzymes (Figure 5E). This result could be in part due to the basal activity presented by PKD in neurons in culture as detected by the kinase autophosphorylation signal phospho-Ser^916^.

### PKD1 activity controls nNOS activation and NO synthesis

**Figure 6:**
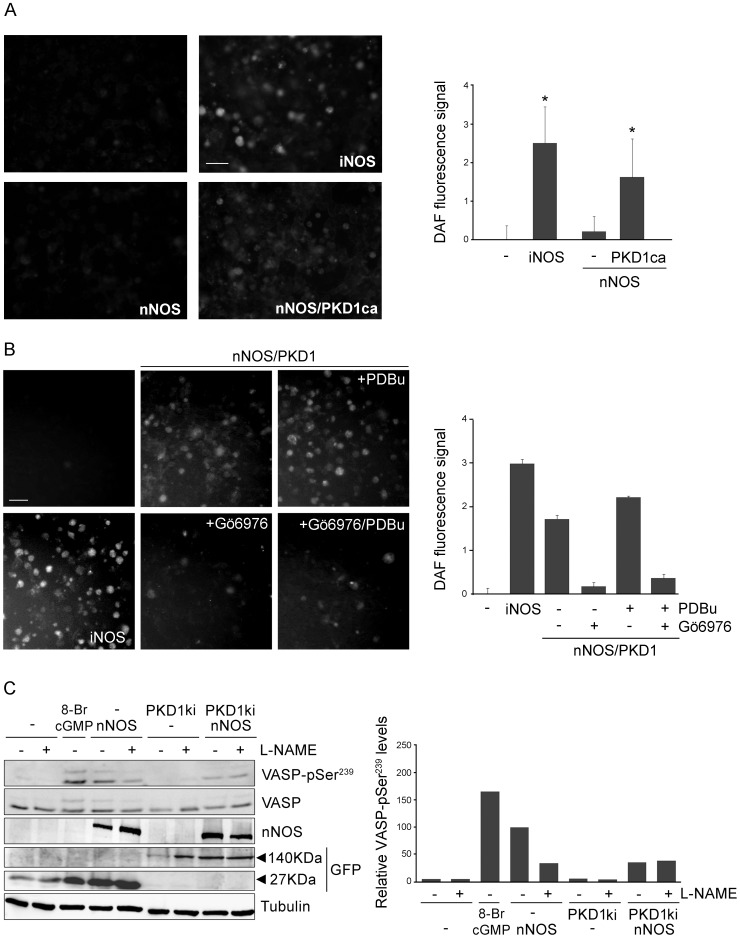
PKD1 activity controls ·NO production and downstream cGMP/PKG signaling. (**A**) COS-7 cells were transfected with full-length wild-type nNOS in the absence or presence of PKD1ca. In a different well, iNOS was also transfected and served as a control of large amounts of released ·NO. 48 h after transfection cells were washed with medium and incubated with 25 µM of the fluorescent ·NO sensor DAF2-DA. Cell-released ·NO was allowed to react with DAF2-DA for at least 4 hours. Subsequently, the monolayer was extensively washed with medium and the fluorescence was detected between 505 and 525 nm using an excitation wavelength of 488 nm. A minimum of three large monolayer fields of over 400 cells were captured. A representative field is shown for each of the four conditions (left panels). Fluorescence was quantified through pixel to pixel intensity determination and signal corresponding to cells transfected with the empty vector was subtracted from each condition to represent the plot on the right. Data are mean ± S.D. for three determinations. *, *p*<0.05 in relation to non-transfected cells. (**B**) COS-7 cells were transfected with full-length wild-type nNOS and wild-type PKD1. In a different well, iNOS was also transfected as a positive control. Two days after transfection cells were incubated with DAF2-DA as before, then preincubated or not with Gö6976 (5 µM) and treated or not with PDBu (200 nM) for 15 min. A representative field for each of the six conditions is shown (left panels) and fluorescence was quantified as above (right panel). (**C**) HEK293T cells were cotransfected with either pEFBOS-GFP vector alone (−) or kinase inactive GFP-PKD1 (PKD1ki) and Myc-nNOS. Cells were pre-treated or not with the nNOS inhibitor L-N^G^-nitroarginine methyl ester (L-NAME; 100 µM) for the last 24 h and 24 h after transfection. Two days after transfection cells were lysed and total lysates were analyzed by immunoblot. Detection of phosphorylated VASP (VASP-pSer^239^) as a doublet of 45 kDa and 50 kDa was used as a measurement of downstream signaling activated by ·NO production. As a positive control, VASP-pSer^239^ phosphorylation was triggered by incubating the cultures with the cGMP homologue 8-Br-cGMP (100 µM) for 30 min. Levels of total VASP, nNOS, GFP and GFP-PKD1ca expression in total lysates are also shown. Note that total VASP appears as a doublet which upper band is absent in unstimulated cells. Signal for α-tubulin was used as loading control. Graph on the right represents the quantification of the immunoblot signals corresponding to the two VASP-pSer^239^ bands (45 kDa plus 50 kDa) normalized to α-tubulin levels and expressed relative to the values obtained in cells expressing nNOS in the absence of L-NAME (arbitrarily assigned a value of 100%). Representative results from three independent experiments are shown. Note that co-transfection of inactive PKD1 abrogates nNOS-induced phosphorylation of VASP-pSer^239^ levels to the same extent as L-NAME inhibitor, indicating that nNOS activation, production of ·NO and stimulation of cGMP/PKG signaling pathway is under the control of PKD activity.

So far our data show that PKD1 activation enhances the formation of a complex with nNOS and PKD1 phosphorylates nNOS at Ser^1412^. It has been reported previously that nNOS activity is regulated through the concerted action of several protein kinases and phosphatases [4], [44], [45]. In fact, various signaling pathways result in the activation of protein kinases (such as Akt/PKB or PKA) that converge in the phosphorylation of Ser^1412^, activation of nNOS and increased ·NO synthesis [31], [46], [47]. Hence, our findings indicate that PKD1 might be a newly identified activatory partner of nNOS. Our next goal was to demonstrate that active PKD1 was in fact stimulating nNOS enzymatic activity and inducing the production of ·NO. As a first approach we used DAF2-DA (4,5-Diaminofluorescein diacetate), a reagent that is used to detect and quantify low concentrations of nitric oxide when loaded into cells [48]. Transfection of COS-7 cells with wild-type nNOS resulted in a modest increase in ·NO synthesis and DAF2-DA fluorescence levels, probably due to the absence of any added calcium ionophores (Figure 6A, quantification graph represented on the right). However, ·NO production increased significantly in cells where nNOS had been cotransfected with a constitutively active mutant of PKD1 (PKD1ca). As a positive control, we transfected COS-7 cells with iNOS, an isoform that binds Ca^2+^/calmodulin irreversibly and induces the release of high amounts of ·NO (Figure 6A). In addition, we also examined the effects of PKD inhibition in ·NO synthesis by co-expressing wild-type nNOS and PKD1 in COS-7 cells. DAF2-DA fluorescence signal obtained in untreated cells or after PDBu stimulation was blocked when cells were pretreated with Gö6976 (Figure 6B, quantification graph represented on the right). These results clearly show that PKD1 activity increases the synthesis of ·NO by nNOS in living cells.

**Figure 7:**
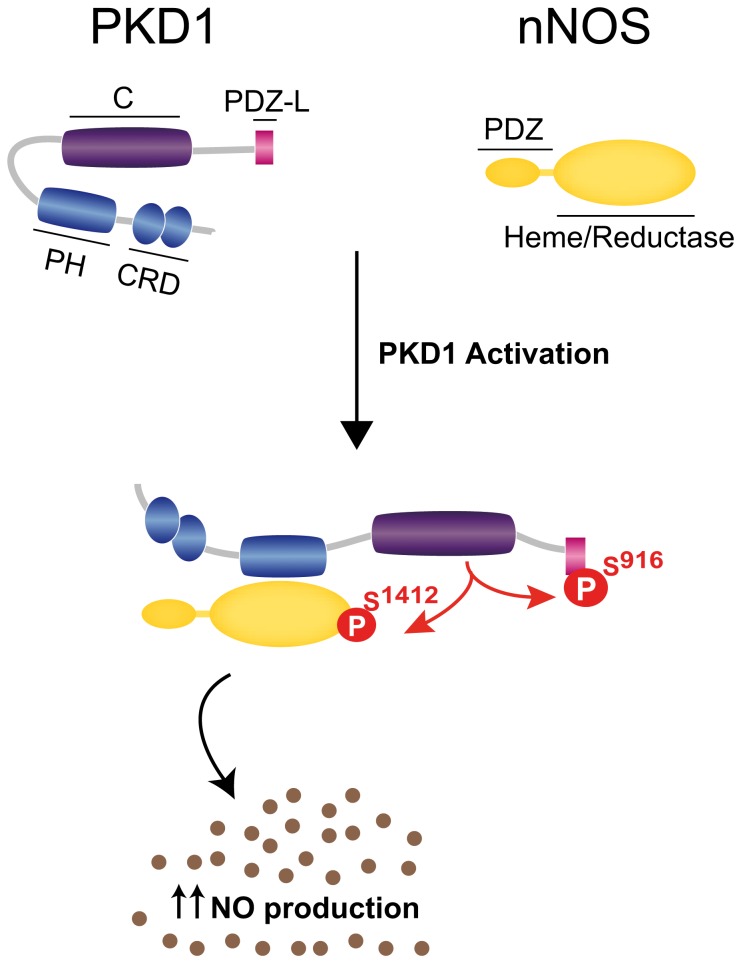
Scheme model of PKD1/nNOS complex formation, nNOS activatory phosphorylarion and NO synthesis. Activation of PKD1 enhances the association of PKD1 with nNOS. The PH domain of PKD1 mediates a direct interaction with nNOS that is independent of PKD1 PDZ-ligand. Active PKD1 autophosphorylates at Ser^916^ and phosphorylates the activatory residue Ser^1412^ within nNOS C-terminal α-helix, leading to the stimulation of nNOS activity and enhancement of ·NO production. CRD, cysteine rich domain; PH, pleckstrin homology domain; C, catalytic domain; PDZ-L, PDZ-ligand.

As a read out of ·NO synthesis we also decided to determine the levels of vasodilator-stimulated phosphoprotein (VASP) phosphorylated at Ser^239^ (VASP-pSer^239^), which has been suggested to represent a biochemical marker of ·NO levels in intact cells [49]. Released ·NO is able to induce cGMP production and protein kinase G activation that ultimately phosphorylates this residue in VASP, an effect prevented by preincubation with NOS inhibitors. Protein kinase G activation promotes VASP phosphorylation mainly in Ser^239^, but also in other two residues [50], [51]. Unphosphorylated VASP and VASP-pSer^239^ migrate in SDS-PAGE gels with an apparent molecular weight of 45 kDa and additional phosphorylations in either one or both of the other residues produces a shift up to 50 kDa. Therefore, depending on the activation of this signaling cascade, the antibody recognizing VASP-pSer^239^ will detect a double band in immunoblot analysis. Importantly, if the phosphorylation of the protein decreases significantly, total VASP will mainly be detected as a single band of 45 kDa. Changes in the intensity of VASP-pSer^239^ band or in the mobility of the protein correlate with the degree of phosphorylation and consequently of the activation/inactivation of this pathway by ·NO. To determine the influence of PKD1 activity on this parameter, we transfected HEK293T cells with Myc-nNOS and kinase inactive GFP-PKD1ki (PKD1ki) (Figure 6C). Before preparing cellular extracts, cells were pre-treated or not with the nNOS inhibitor L-N^G^-nitroarginine methyl ester (L-NAME; 100 µM) for the last 24 h. As a positive control, we triggered VASP-pSer^239^ phosphorylation by incubating the cultures with the cGMP homologue 8-Br-cGMP (100 µM) for 30 min. The immunoblot image and its quantification analysis showed that VASP-pSer^239^ levels were almost undetectable in untreated and that the doublet signal only appeared after 8-Br-cGMP treatment (Figure 6C). Regarding total VASP, in 8-Br-cGMP stimulated cells a doublet was clearly visible which upper band was hardly detectable in unstimulated cells. When nNOS was expressed VASP-pSer^239^ signal was clearly potentiated, an effect that was partially blocked by the inhibitor L-NAME. Transfection of kinase inactive PKD1 had no effect on VASP-pSer^239^ or total VASP, being their signal very similar to control cells (Figure 6C). Noteworthy, when inactive PKD1ki was co-expressed with nNOS, VASP phosphorylation at Ser^239^ was significantly decreased compared with that of cells expressing nNOS alone (Figure 6C, quantification graph represented on the right) and similar to that obtained in cells pretreated with L-NAME. The reduction in the signal of VASP-pSer^239^ and the lack of effect of nNOS inhibitor L-NAME that confers kinase inactive PKD1 demonstrates that the stimulation of this pathway is greatly hampered when PKD1 activity is compromised. Importantly, we also detected an increase in VASP-pSer^239^ signal as readout of ·NO release and signaling in mature neuronal cultures after NMDAR stimulation, an effect blocked by inhibiting PKD with Gö6976 (Figure 5D). In summary, our data demonstrate that there is a direct correlation between PKD1 activity, nNOS phosphorylation and activation, and ·NO production (see model in Figure 7).

## Discussion

Among the three enzymes involved in ·NO synthesis (eNOS, nNOS and iNOS), the neuronal isoform nNOS is the only one bearing a PDZ domain. It is widely accepted that PDZ domains present selective interaction with specific PDZ-ligands. Particularly, the PDZ domain of nNOS was proposed long time ago to display a preference for PDZ-binding motifs bearing acidic residues at -2 or -3 position [6], [7]. The discovery by our group of a PDZ-ligand autophosphorylated in Ser^916^ at -2 position in active PKD1, therefore presenting a negatively charged phosphorylated residue at this site [26], prompted us to hypothesize that this motif could be interacting directly with the PDZ domain of nNOS. We demonstrate here there is a spatial and physical association of PKD1 and nNOS that is potentiated by activation of the kinase. Unexpectedly, our experiments performed in yeast and mammalian cells, show that the association of these two enzymes occurs independently of the PDZ-ligand of PKD1 and the phosphorylation state of Ser^916^ within this motif. Instead, the PH domain of PKD1 is absolutely required for its association with nNOS, mediating a direct interaction of both enzymes. Several years ago we discovered that the PH domain of PKD1 is autoinhibitory since point mutations or complete deletion of this domain render a constitutively active kinase [36]. Despite of its highly active state, and the consequent autophosphorylation at Ser^916^ within the PDZ-ligand, PKD1 mutant lacking the PH domain (PKD1^ΔPH^) is unable to associate with nNOS. Importantly, α-syntrophin, which interaction with nNOS through its PDZ domain was first identified [14], bears a PH domain needed to target nNOS to the sarcolemma *in vivo*, in addition to its PDZ domain [52], [53]. These observations suggest that there might be a common molecular mechanism by which PH domains may play a critical role in the regulation of nNOS associations with protein complexes and/or subcellular compartments.

The PH domain of PKD1 was first identified to mediate direct interactions preferentially with protein kinase C (PKC) novel isoforms, PKCη and PKCε [35]. These PKCs participate in the classical pathway of PKD1 activation (induced by phorbol esters or diacylglycerol production - downstream membrane receptor's activation) by phosphorylating activation loop Ser^744^ and Ser^748^ [54], which in turn results in a release of autoinhibition by the PH domain [55]. PKCδ, another member of the novel PKC subfamily, participates in oxidative stress-induced PKD activation by molecular mechanisms that involve an initial activation of Abl and Src tyrosine kinases [56], [57]. In this alternative pathway, Src-mediated Abl activation leads to Tyr^463^ phosphorylation within PKD1 autoinhibitory PH domain and provokes a molecular switch that allows Src-mediated Tyr^95^ phosphorylation, the formation of a complex with PKCδ facilitating activation loop phosphorylation and correlated PKD1 activation [56], [57]. Despite the pathway involved, a conformational change and a relief of PH domain autoinhibition accompany PKD activation. This novel open conformation may present a more accessible PH domain and favor the association of active PKD with different protein complexes, as we show here to occur with nNOS. Similarly to nNOS, the kinase activity and the PH domain of PKD are critical for apoptosis signal regulating kinase 1 (ASK1) interaction and activation [58]. However, we show here that PKD directly phosphorylates and activates nNOS whereas there are no evidences for ASK1 being a PKD substrate or of stimulation of ASK1 activity by direct PKD phosphorylation.

Despite the list of PKD substrates is increasing, very little is known about the biological significance of their association to the kinase. For most PKD substrates, like Hsp27 [59], troponin [60], snail [61], slingshot [62], RIN1 [63], [64], CERT [65], oxysterol binding protein [66] or sphingosine kinase [67], there are not association studies available. In the case of rhotekin [68] and phosphatidylinositol-4 kinase III-β [69] the association studies gave negative results. Interestingly, some PKD substrates have been shown to associate with the kinase, such as Kidins220 [20], HDAC5 [70], E-cadherin [71], β-catenin [72], CREB [73] and cortactin [41]. However, the effect of PKD activation on substrate association was only specifically addressed before for Kidins220 [20] and HDAC5 [70] that form complexes with the kinase independently of its activation state. Therefore, nNOS is the first identified substrate which interaction with PKD is clearly enhanced after activation of the kinase.

In addition, we demonstrate here that active PKD1 phosphorylates nNOS in the activatory Ser^1412^ *in vitro* and *in vivo* in living cells, stimulating its enzymatic activity and increasing ·NO production. In this context, it must be mentioned that this is an atypical site for PKD phosphorylation. This kinase usually recognizes a consensus motif presenting a hydrophobic residue such as Leu/Val/Ile at -5 position and Arg/Lys at -3 position referred to the phosphorylatable Ser residue [38]. Remarkably, nNOS displays Arg residues at both positions (RLRSES^1412^). Regardless of this fact, our data show that PKD1 is an activatory partner of this isoform by phosphorylating Ser^1412^. This residue is part of a motif positioned at the very C-terminal end of the reductase module and recent crystallographic data indicate that it adopts a helical conformation [39]. This α-helix is known to function as a physical “lid” in the reductase domain that impedes proper electron transfer [74], [75]. The phosphorylatable oxygen atom of Ser^1412^ is directed toward the negatively charged flavin mononucleotide-binding domain residues Glu^916^ and Asp^918^ [39]. It has been suggested that this arrangement thus rationalizes a mechanism for phosphorylation-induced NOS activation. The electrostatically-induced conformational change would be then mediated by the repulsion of the negative charge of the newly added phosphate group. Hence, phosphorylation of nNOS at Ser^1412^ activates electron transfer from the reductase domain towards the oxygenase domain of nNOS thus augmenting ·NO synthesis and cGMP formation [39].

Similarly, it has been reported that nNOS is also phosphorylated on Ser^1412^ by Akt/PKB [31], [76], cyclic AMP-dependent protein kinase (PKA) [46] and AMP-activated protein kinase (AMPK) [47] in neurons and in skeletal muscle. Not only in nNOS, but also in eNOS, the equivalent serine residue (Ser^1179^ and Ser^1177^ in bovine and human eNOS, respectively), located at the C-terminus (presumably an α-helix as well), was early recognized as a phosphorylation site. This serine residue is immersed within a consensus Akt/PKB-dependent phosphorylation consensus sequence (RXRXX(S/T)X, herein RLRSESI in nNOS and RIRTQSF in eNOS). We and other authors reported the phosphorylation of eNOS by Akt/PKB [77]–[79] and AMPK [80] both *in vitro* and in a cellular environment. Furthermore, the activity of at least six protein kinases (Akt/PKB, AMPK, PKA, cGK-I/PKG, Chk1 and CaMKII converge on the same eNOS activatory Ser^1179^, and in all cases protein phosphorylation correlates with activation and increased ·NO synthesis (for a recent review see Dudzinski and Michel [81]). In the case of eNOS, mimicking the phosphorylation of Ser^1179^ by introducing an acidic residue in recombinant purified enzymes directly enhances enzyme activity and alters the sensitivity of the enzyme to Ca^2+^, rendering its activity maximal at sub-physiological concentrations of this cation [82]. Importantly, we have also identified that PKD1 induces eNOS phosphorylation on Ser^1179^ *in vitro* and in endothelial cells stimulated with vascular endothelial growth factor (unpublished data).

Our data also show that activated PKD1 and substrate nNOS colocalize and are able to co-immunoprecipitate. This is not unprecedented, since the homologous eNOS can be co-immunoprecipitated with several protein kinases, such as Akt/PKB [83], AMPK [84] and Chk1 [85], known to phosphorylate the equivalent serine at the activatory C-terminus upon activation. The proximity of activated PKD1 and nNOS observed in transfected neural PC12 cells differentiated with nerve growth factor suggests that these two enzymes might be together at neuronal post-synaptic densities. Supporting this possibility, we also show that the stimulation of glutamate NMDAR with the specific agonist NMDA in primary cortical neurons in culture results in an activation of PKD1 that parallels nNOS phosphorylation at Ser^1412^ which is blocked after pharmacological inhibition of PKD. Other authors have reported previously that protein kinase Akt/PKB activation in response to NMDARs stimulation also results in nNOS phosphorylation at Ser^1412^ and increases its enzymatic activity [31]. It would be of high interest to follow this line of research in the future in order to establish the role of PKD1 activation downstream NMDAR stimulation and its relation to ·NO production both under physiological and pathological conditions. It is well established that whereas the small quantities of nitric oxide formed during synaptic transmission modulate neuronal signaling, excess of nitric oxide mediates neurotoxicity in pathological situations, such as ischemic stroke or neurodegeneration. Hence, since little is known about the various kinases that regulate nNOS function *in vivo*, PKD inhibitors might be useful drugs in cases of nitric oxide associated neurotoxicity.

In conclusion, herein we reveal that PKD1 interacts with nNOS and phosphorylates Ser^1412^ enhancing this way nNOS activity and ·NO production. Considering that the corresponding serine in eNOS is subjected to similar mechanisms of phosphorylation-dependent activation, PKD emerges as a common regulator of the enzymatic activity of constitutive NOS isoforms and ·NO synthesis. These novel findings add to the list of biological relevant roles of PKD a crucial one in the regulation of ·NO synthesis and the plethora of physiological and pathological processes where this mediator is involved.

## Supporting Information

Figure S1 **Association of PKD2 with nNOS.**
(TIF)
