# Supplementary figures and images for: Protein Kinase D Interacts with Neuronal Nitric Oxide Synthase and Phosphorylates the Activatory Residue Serine1412
Source: PLoS One. 2014 Apr 16;9(4):e95191. doi: 10.1371/journal.pone.0095191 (PMC3989272; doi:10.1371/journal.pone.0095191)

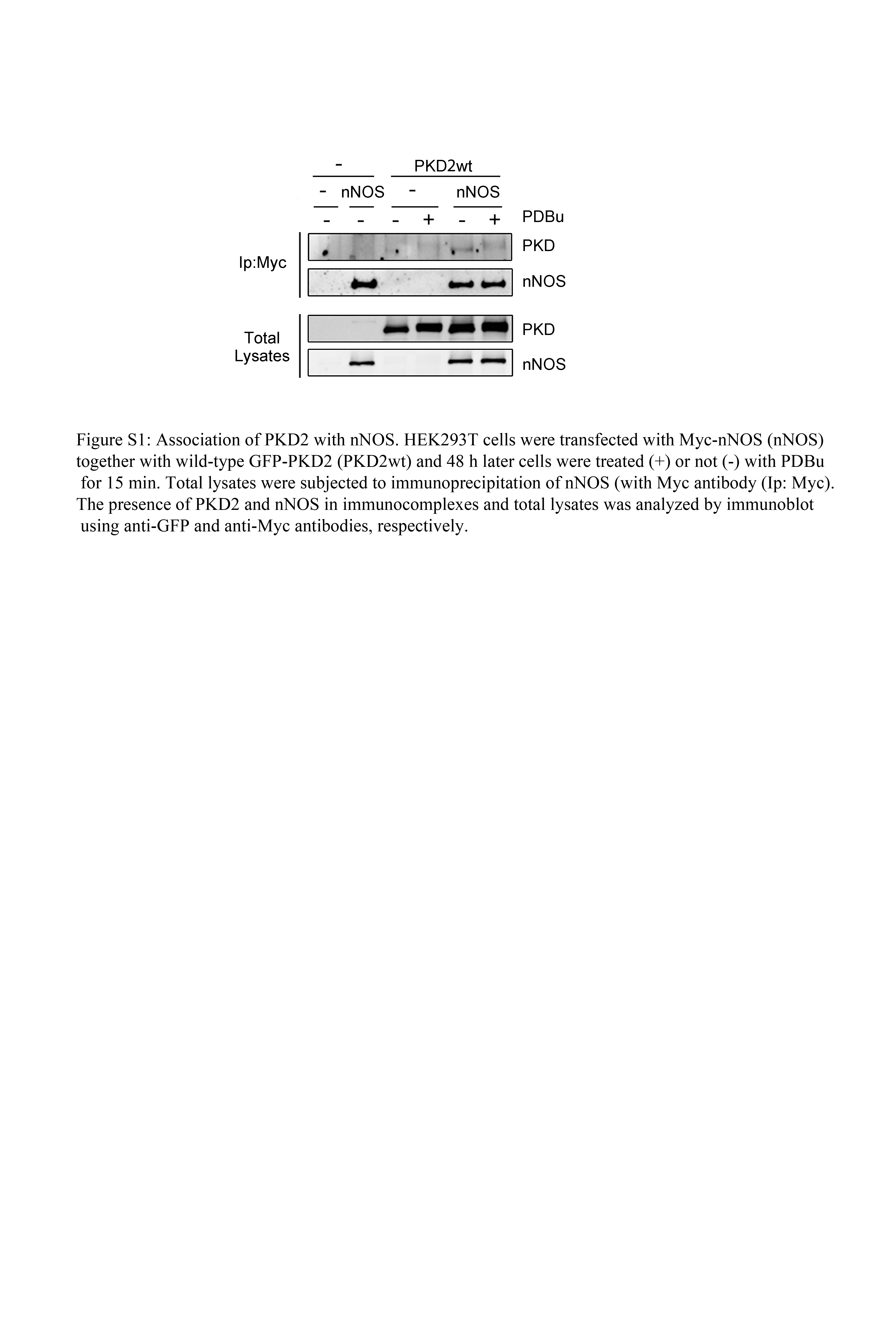

Supplement: Figure S1 — Association of PKD2 with nNOS. (TIF) [file pone.0095191.s001.tif]
